# CHARACTERIZING THE PATHWAYS UNDERLYING THE ASSOCIATION BETWEEN PSYCHOLOGICAL WELL-BEING AND HEALTH

**DOI:** 10.1093/geroni/igz038.2996

**Published:** 2019-11-08

**Authors:** Eric S Kim, Anthony Ong

**Affiliations:** 1 Harvard T.H. Chan School of Public Health, Boston, United States; 2 Cornell University, Ithaca, New York, United States

## Abstract

As populations age, identifying factors that foster the maintenance of health is crucial for improving the health and well-being of older adults. Yet, most psychological, biomedical, and public health efforts have focused on reducing harmful risk factors. While the risk management approach has contributed greatly to prevention and treatment programs, our aging society continues to grapple with the steadily rising tide of chronic conditions. Expanding the focus to include upstream, health-promoting psychosocial assets may help inform a more comprehensive response effort. Mounting research suggests that different dimensions of psychological well-being are uniquely associated with reduced risk of chronic conditions. However, the mechanisms underlying these associations remain understudied. This symposium presents 4 studies evaluating potential mechanisms. The first talk presents research evaluating how a spouse’s level of optimism may be uniquely associated with an individual’s cognitive health over time (above and beyond that own individual’s level of optimism). A second talk, draws upon a multi-burst daily diary study and focuses on affective stress response as a potentially modifiable target that could explain the health benefits of optimism. A third talk evaluates how baseline levels purpose in life might be associated with repeated measures of five key health behaviors over time. A fourth talk discusses results from a longitudinal-burst daily diary study determining the reciprocal relationships among optimism, pain interference, and goal-directed activity among older women who experience pain. Overall, these studies add to the growing research on psychological well-being and physical health by providing evidence around potential biobehavioral pathways.

